# Attitude toward dementia and preferences for diagnosis in Japanese health service consumers

**DOI:** 10.1186/s12913-021-06381-9

**Published:** 2021-05-03

**Authors:** Hikaru Oba, Teruyuki Matsuoka, Yuka Kato, Rochelle Watson, Elise Mansfield, Rob Sanson-Fisher, Jin Narumoto

**Affiliations:** 1 Graduate School of Health Sciences, Hirosaki University, 66-1, Hon-cho, Hirosaki-city 036-8564 Aomori, Japan; 2 Department of Psychiatry, Graduate School of Medical Science, Kyoto Prefectural University of Medicine, 465 Kajii-cho, Kawaramachi-Hirokoji, Kamigyo-ku 602-8566 Kyoto, Japan; 3 Health Behaviour Research Collaborative, School of Medicine and Public Health, Faculty of Health and Medicine, University of Newcastle, NSW 2308 Callaghan, Australia; 4 Priority Research Centre for Health Behaviour, University of Newcastle, NSW 2308 Callaghan, Australia; 5 Hunter Medical Research Institute, NSW 2305 New Lambton Heights, Australia

**Keywords:** Dementia, Early diagnosis, Feared disease, Advance care planning, Decision making

## Abstract

**Background:**

Being diagnosed with dementia is a confronting experience for any individual and their caregiver. However, a diagnosis provides opportunity for future preparation for management of the condition. This study investigated attitudes toward dementia and preferences for diagnosis among a sample of health service consumers in Japan.

**Methods:**

Participants were patients or accompanying support persons (*n* = 217) who visited the specialty outpatient clinic of four hospital departments. The survey was conducted using an iPad with answers sent automatically to a secure server. The survey included items about the participants’ most feared diseases and the reasons behind those fears, estimates of dementia prevalence in Japan, and preferences regarding a diagnosis of dementia and the reasons for their preference.

**Results:**

The most feared disease was cancer (43.8 %), followed by dementia (18 %). Those selecting dementia most commonly reported practical, emotional and social impacts as the reasons why they most feared this condition. Almost all participants preferred to know the diagnosis of dementia as soon as possible for themselves, with significantly fewer preferring their spouse to know as soon as possible if they had dementia (95.9 % for self vs. 67.5 % for partner/spouse, *p* < 0.001). On average, participants estimated that 18.1 % of Japanese people are diagnosed with dementia by age 65, while they thought that 43.7 % of Japanese people are diagnosed with dementia by age 85.

**Conclusions:**

The findings highlight a need for community education about the significant impacts of dementia on the lives of individuals and their caregivers. People were more reluctant for their spouse to receive a diagnosis as soon as possible if they had dementia. Physicians should sensitively disclose diagnosis and ensure they involve both the patient and their relatives in discussions about diagnosis disclosure.

## Background

The World Health Organization (WHO) reported that there are around 50 million people with dementia worldwide [1]. Japan has the highest elderly rate (28.1 %) in the world, with the number of people living with dementia estimated at 4.62 million people in 2012 [2–4]. The report also showed that the prevalence of dementia was 1.5 % for those aged 65–69 years, and 27 % for those aged 85 years [2].

In Japan, early diagnosis of dementia is sometimes called “early despair” by people who are suspected of dementia and their caregivers because the treatment effect is limited. However, recent studies have emphasized the benefits of an early or timely diagnosis of dementia. Earlier diagnosis provides time to consider medical, psychological, financial, legal, and practical implications of having a dementia diagnosis [5, 6].

Some barriers that prevent an early diagnosis of dementia include a lack of support, stigma, and financial constraints among doctors, patients, and caregivers [7, 8]. While the most common cause of death in the world is ischemic heart disease, death due to dementia is increasing particularly in high-income countries [9]. As many people fear suffering from dementia, negative emotions associated with a suspected case of dementia may delay the timing of diagnosis [8]. Some systematic reviews showed that approximately 60 % of people never receive a formal diagnosis of dementia [10, 11].

Approaches to disclosing the diagnosis are also variable. Many professionals discuss the diagnosis with relatives but not with people who have dementia [11, 12]. One study showed that a diagnosis was more likely to be disclosed to people who had more severe cognitive impairments [12]. This result suggests that general practitioners may have difficulty disclosing early stage diagnoses due to the perceived negative impact on their patients [7, 11, 13]. However, another study found that following diagnosis, there was no increase in depression and interestingly a reduction in anxiety, suggesting physicians’ fears about negative reactions to a diagnosis may be unfounded [14]. Understanding individuals’ willingness to receive a diagnosis if they had dementia and the reasons for their preference will provide important information to guide physicians’ approaches to diagnosis disclosure.

Previous studies have examined the preferences for disclosure of dementia diagnosis among people living in the community. In the Japanese sample, 76–80 % of people preferred to be diagnosed with dementia when they first noticed symptoms [15], while studies conducted in other countries found larger proportions of people preferred to be diagnosed when symptoms were first noticed [11, 16–19]. Understanding Japanese health care consumers’ knowledge and fears regarding dementia and preferences for diagnosis disclosure can provide important information about how physicians can support those who are diagnosed with dementia.

## Methods

### Aims

The present study investigated among health service consumers in Japan: (1) fear of dementia relative to other health conditions and the reasons for this; (2) knowledge about the prevalence of dementia; and (3) preference for dementia diagnosis and the reasons for this.

### Design and setting

The survey was conducted from September 2017 to March 2019 in the psychiatry, otolaryngology, endocrinology and metabolism, and cardiovascular medicine outpatient clinics of a major teaching hospital in an urban area in Japan. Patients attending a clinic appointment or their accompanying support persons aged 18 years over participated in the survey. Primary physicians consulted the medical chart and excluded individuals who were diagnosed with dementia or showed severe physical or psychiatric symptoms. Primary physicians also asked their patients and accompanying support persons whether they had time to hear the explanation about the survey when they visited the outpatient clinic. Patients were assured that their decision to participate would in no way affect their medical care. Some people declined to hear the explanation due to time restrictions. A research assistant obtained informed consent after a full explanation of the survey. The data were collected by a web-connected iPad which automatically sent participant responses to a secure server at the University of Newcastle. Commencement of the survey was taken as participants’ voluntary consent to participate. QuON, a web-based survey software application was used to manage the survey [20].

### Measures

The survey items were adapted from two previous Australian studies (17; one yet unpublished) and the questions regarding the prevalence rate of dementia were added.

### Socio-demographics

Age, sex, education, marital status, and acquaintance with someone who was diagnosed with dementia were collected.

### Feared disease and the reasons

We asked participants to report their most feared disease from the following: coronary heart disease, dementia, cerebrovascular disease (CVD), cancer, chronic obstructive pulmonary disease, diabetes, and heart failure. Participants were also asked to select up to the three reasons why they fear the selected disease (shortened length of life, emotional impact (e.g. depression, stress, loss of sense of ‘self’), social impact (e.g. stigma, loss of respect from friends, feeling isolated), practical issues (e.g. loss of independence, needing help to do daily tasks family would need to care for me), financial strain (e.g. would be unable to work, or pay for treatments), physical symptoms and side-effects (e.g. pain, fatigue, vomiting), legal issues (e.g. preparing wills, appointing someone to make decisions on my behalf), and other (Please specify).

### Recognition of the prevalence rate of dementia

To gauge participants knowledge of the prevalence of dementia, they were asked two questions in the survey: “Of 100 Japanese, how many do you think will have been diagnosed with dementia by age 65?”, and “Of 100 Japanese, how many do you think will have been diagnosed with dementia by age 85?”. Participants typed in the number they thought was correct.

### Preferences for the timing of diagnosis disclosure

Participants were presented with the situation: “Imagine you see your doctor and test results show that you have dementia. Given there is no cure, when would you want your doctor to tell you that you have dementia?”. Participants selected one response from four possible options: As soon as possible, I would not want to know until my symptoms got worse or made me really worried, Only when my family thought it was necessary to tell me, and I would not want to know my diagnosis at all.

We also asked the reasons for their preference. Where participants answered ‘as soon as possible’, they were asked to select from the following reasons: ‘Make the most of life (e.g. ‘bucket list’)’, ‘Collect memories’, ‘Work on my relationships’, ‘Tell loved ones my situation’, ‘Come to terms with the diagnosis’, ‘Be involved in decisions about my future care’, ‘Make financial arrangements for with family’, ‘Access treatments and support’ and ‘Other (Please specify)’.

Where participants answered any of the other three remaining options, they were asked to select from the following reasons: ‘There is no cure / no benefit of knowing’, ‘Risk of incorrect diagnosis’, ‘It would cause me to feel depressed’, ‘Fear that people would treat me differently’, ‘To avoid unnecessary worry’, ‘To avoid putting strain on my relationships’, ‘Fear that knowing the diagnosis might make symptoms progress faster’,’ So I could live normally for as long as possible’, ‘Other’. Furthermore, participants who had a partner or spouse were also asked the preference in the imaginary situation where their partner or spouse was diagnosed with dementia. In this case, participants were asked when they would like their spouse to know if they had a diagnosis. The same response options were provided with wording changes as appropriate to refer to the spouse.

### Statistical analysis

Descriptive statistics of all measures were analyzed. Pearson’s chi-squared test was conducted to compare the proportion of most feared disease by age group (aged 65 years over or under 65 years) and the preference for timing of diagnosis disclosure to self or partner/spouse. Comparison of the reasons why cancer and dementia were selected as most feared was also conducted. Fisher’s exact test was used when cell counts were low for some response options. The preference for timing of diagnosis was transformed into the binominal data of “as soon as possible” and “waiting” because few participants chose the latter option. The analysis was conducted using R 3.4.3 [21].

## Results

There was no significant difference in survey responses between patients and support persons, therefore data from these groups are reported together. All eligible participants who were informed about the study agreed to participate. A total of 217 participants (134 male and 83 female) were analyzed. Participants were attending psychiatry (*n* = 30, 13.8 %), otolaryngology (*n* = 34, 15.7 %), endocrinology and metabolism (*n* = 101, 46.5 %) and cardiovascular medicine (*n* = 52, 24.0 %) appointments. The mean age of the participants was 64.4 years (*SD* = 13.0, *Min-Max* = 19–91). Just over three quarters reported they had a partner or spouse (*n* = 163, 77.3 %). Half of participants (*n* = 110, 50.7 %) knew a person who was diagnosed with dementia but most of them were not providing care to that person (*n* = 94, 85.5 %). Socio-demographic characteristics and acquaintance with people having dementia were shown in Table 1.

**Table 1:** Descriptive statistics of participants (*n* = 217)

	*n*	*%*
Age	64.4 ± 13.0	
Sex		
Male	134	61.8
Female	83	38.2
Partner or spouse		
Yes	163	77.3
No	48	22.7
Missing value	6	
Education		
Elementary School	0	0
Junior High School	22	10.2
High School	80	37
Trade or Vocational Training	35	16.2
Bachelor Degree	73	33.8
Postgraduate Degree	6	2.8
Missing value	1	0.5
Marital Status		
Single/Never married	24	11.1
Married/Living with partner	152	70
Separated or Divorced	21	9.7
Widowed	20	9.2
Know the person who was diagnosed dementia		
Yes	110	50.7
No	107	49.3
Relationship with person with dementia (*n* = 110)		
Partner/spouse	5	4.5
Parent	13	11.8
Grandparent	10	9.1
Brother or sister	6	5.5
Friend	20	18.2
Other	56	50.9
Carer of person with dementia (*n* = 110)		
Yes	16	14.5
No	94	85.5

Cancer (*n* = 95, 43.8 %) was selected as the most feared disease, followed by dementia (*n* = 39, 18 %) and CVD (*n* = 39, 18 %). Chi-square test showed that a larger proportion of participants aged 65 years or over reported dementia as their most feared disease (*n* = 27, 69.2 %) compared to those under 65 years (*n* = 12, 30.8 %, *p* = 0.016). There was no difference by age for any other disease. Table 2 shows the comparison of the reasons for selecting cancer and dementia as the most feared disease. Participants who selected dementia as their most feared disease more commonly reported the practical impact (87.2 % vs. 54.7 %, ${\chi }^{2}$ = 12.7, *p* < 0.001), emotional impact (61.5 % vs. 30.5 %, ${\chi }^{2}$ = 11.1, *p* = 0.001), social impact (51.3 % vs. 2.1 %, ${\chi }^{2}$ = 48.7, *p* < 0.001), and legal impact (20.5 % vs. 6.3 %, odds ratio = 0.264, *p* = 0.026 in Fisher’s exact test) as the reasons they feared dementia compared to those who selected cancer.

**Table 2:** Comparison of the reasons with cancer and dementia (*n* = 217)

	Cancer (*n* = 95)		Dementia (*n* = 39)		*p*
	*n*	*%*	*n*	*%*	
Shortened length of life	47	49.5	0	0	< 0.001
Emotional impact	29	30.5	24	61.5	0.001
Social impact	2	2.1	20	51.3	< 0.001
Practical issues	52	54.7	34	87.2	< 0.001
Financial strain	33	34.7	10	25.6	0.306
Physical symptoms and side-effects	59	62.1	1	2.6	< 0.001
Legal issues^a^	6	6.3	8	20.5	0.026
Others^a^	8	8.4	3	7.7	1.000

^a^Fisher’s exact test. Proportions do not add to 100 % as participants could select up to three reasons

On average, participants estimated that 18.1 % (*SD* = 15 %, *Min-Max =* 1–80 %) of Japanese people have dementia by age 65, while they estimated that 43.7 % (*SD* = 22.6 %, *Min-Max =* 3- 100 %) of Japanese people have dementia by age 85. These are over-estimates compared to actual prevalence rates in Japan of 1.5 % for people aged 65–69 years and 27 % of those aged 85 years.

Descriptive statistics of participants’ preference for timing of dementia diagnosis and the reasons for their preference are shown in Table 3. Almost all participants (*n* = 208, 95.9 %) hoped to be diagnosed as soon as possible if they had dementia. For participants who responded they would like to know their diagnosis as soon as possible, ‘Come to terms with the diagnosis’ (*n* = 141, 67.8 %) was the most frequent reason for this preference, followed by ‘Access treatments and support’ (*n* = 125, 60.1 %), and ‘Be involved in decisions about my future care’ (*n* = 105, 50.5 %). Participants who did not answer ‘As soon as possible’ (*n* = 9, 4.1 %) selected ‘Avoid unnecessary worry’ (*n* = 6, 66.7 %) or ‘So I could live normally for as long as possible’ (*n* = 6, 66.7 %) followed by ‘It would cause me to feel depressed’ (*n* = 4, 44.4 %), and ‘To avoid putting strain on my relationships’ (*n* = 4, 44.4 %).

**Table 3:** Preferences for timing of diagnosis disclosure (*n* = 217)

	Self		Partner/Spouse		*p*
	*n*	*%*	*n*	*%*	
As soon as possible	208	95.9	110	67.5	< 0.001
Waiting	9	4.1	53	32.5	
Got worse	3	33.3	12	22.6	
Necessary	4	44.4	24	45.3	
Not know at all	2	22.2	17	32.1	
Reasons of the decision^a^					
As soon as possible^b^	(*n* = 208)		(*n* = 110)		
Make the most of life (e.g. ‘bucket list’)	97	46.6	62	56.4	0.099
Collect memories	50	24.0	39	35.5	0.031
Work on my/their relationships	85	40.9	41	37.3	0.533
Tell loved ones my/their situation	79	38.0	41	37.3	0.901
Come to terms with the diagnosis	141	67.8	74	67.3	0.926
Be involved in decisions about my/their future care	105	50.5	58	52.7	0.703
Make financial arrangements for with (their) family	65	31.3	31	28.2	0.571
Access treatments and support	125	60.1	65	59.1	0.862
Other	17	8.2	4	3.6	0.121
Waiting^c^	(*n* = 9)		(*n* = 53)		
There is no cure / no benefit of knowing	3	33.3	9	17.0	0.356
Risk of incorrect diagnosis	0	0	5	9.4	1.000
It would cause me/them to feel depressed	4	44.4	36	67.9	0.259
Fear that people would treat me/them differently	1	11.1	11	20.8	0.675
To avoid unnecessary worry	6	66.7	39	73.6	0.696
To avoid putting strain on my/their relationships	4	44.4	11	20.8	0.201
Fear that knowing the diagnosis might make symptoms progress faster	2	22.2	17	32.1	0.709
So I/they could live normally for as long as possible	6	66.7	40	75.5	0.683
Other	0	0	2	3.8	1.000
It would make them feel bad about themselves	N/A	N/A	18	34.0	

^a^Proportions do not add to 100 % as participants could select more than one response, ^b^ Chi-square test, ^c^ Fisher’s exact test

In the scenario where their partner or spouse are diagnosed with dementia, a majority of participants still preferred their spouse to know their diagnosis as soon as possible (*n* = 110, 67.5 %). However, this proportion was significantly lower than the proportion that would like to know about their own diagnosis as soon as possible (${\chi }^{2}$ = 52.8, *p* < 0.001). Similar to the reasons for preferring to know the diagnosis for oneself, the most frequently selected reasons for wanting their spouse to know as soon as possible were to ‘Come to terms with the diagnosis’ (*n* = 74, 67.3 %), ‘Access treatments and support’ (*n* = 65, 59.1 %), and ‘Be involved in decisions about their future care’ (*n* = 58, 52.7 %). The reason ‘collect memories’ was the only response option that was selected by a significantly different proportion of people when the scenario referred to themselves compared to a spouse (24 % for self vs. 35.5 % in partner/spouse, ${\chi }^{2}$ = 4.65, *p* = 0.031). The most frequently reported reasons for participants preferring that their spouse not be told a diagnosis as soon as possible (*n* = 53, 32.5 %) were ‘So they could live normally for as long as possible’ (*n* = 40, 75.5 %), ‘To avoid unnecessary worry’ (*n* = 39, 73.6 %), and ‘It would cause them to feel depressed’ (*n* = 36, 67.9 %).

## Discussion

The current study investigated knowledge and attitudes toward dementia, and the preferences for disclosure of a dementia diagnosis in Japan. The prevalence rate of dementia was overestimated both at aged 65 and 85 years, and many people feared dementia due to the emotional, social, practical, and legal implications of a diagnosis. Although almost all participants wanted to know the diagnosis of dementia as soon as possible for themselves, far fewer wanted this for their partner or spouse. The present findings provide suggestions for physicians in terms of disclosure of dementia diagnosis to patients or their relatives.

The most feared disease was not dementia but cancer. This result might reflect the statistics in Japan that malignant neoplasms are the most frequent cause of death [3]. Dementia followed cancer as the most feared disease, and was more likely to be selected by participants aged 65 years over. As aging is the greatest risk factor of dementia [22], young people may feel that dementia is a distant future event. With respect to the reasons for dementia being feared, the practical impacts, emotional impacts, and social impacts were more frequently reported than the physical symptoms or shortened length of life. In Japan, the estimated lifetime risk of dementia aged 60 years and over was 64.8 % for women and 40.8 % for men, which was higher rate than the result of other population studies [23]. Since dementia, such as Alzheimer’s disease, is not only characterized by slow progression but also is a common disease for Japanese people, they may be more concerned with the socio-psychological impact than the biological impact. The results suggest people may be unaware that the later stages of dementia can cause symptoms such as incontinence and reduced mobility, and that dementia can reduce one’s life expectancy. Physicians should discuss the disease process and available support systems with patients or their support persons to help to alleviate the socio-psychological impact of dementia when they make a diagnosis. The results also emphasize the need for other health care practitioners, such as psychologists, to contribute to the provision of socio-psychological support after the diagnosis disclosure in order to manage fear associated with the condition.

Although the prevalence of dementia at 65–69 years is 1.5 % and at 85 years is 27 % in Japan [2], participants overestimated the prevalence of dementia by ages 65 and 85. This might be a bias caused by the questionnaire survey method. However, previous studies that have reported the association between knowledge and attitude to people with dementia found community members have less knowledge than people working in dementia care [24, 25]. Previous research has also reported that many in the general public, and even carers and healthcare practitioners, perceive that dementia is a part of normal aging [26]. Our finding suggests the necessity of education or training to improve their dementia knowledge in the community. Moreover, given these estimates, it is perhaps surprising that more participants did not select dementia as their most feared disease. It may be of interest to compare estimated prevalence of dementia with other chronic conditions in future studies.

Almost all participants (95.9 %) wanted to know their diagnosis as soon as possible when they were suspected of having dementia, which is consistent with a previous studies [16–19]. On the other hand, fewer participants (67.5 %) preferred that their partner or spouse be told their diagnosis as soon as possible. The proportion is also lower than that found in other studies in Australia (88 %), Korea (97 %) and Taiwan (76 %) [17–19] The preference not to tell the diagnosis to the partner or spouse may be explained by negative attitudes towards dementia among a significant proportion of the Japanese community. Caregivers’ shame associated with dementia causes stigma to people with dementia such as concealing, shunning, and silencing [27], with approximately 40–50 % of Japanese people having a prejudice that dementia is a shameful disease [28]. This finding might be a reflection of society’s feelings towards people with dementia in Japan. The difference may also help explain why some family members may delay seeking further assessment for possible symptoms of dementia, and why physicians may be reluctant to deliver a diagnosis [7].

There are several limitations in the current study. First, all participants had visited the specialty outpatient clinic of four departments in a hospital, and so were not representative of healthy community members. We did not ask about pre-existing diseases besides dementia that may have affected responses to the questions about feared diseases. Second, questions asked about hypothetical situations. It is unclear whether participants would have the same preference if they were actually experiencing symptoms of dementia. Finally, as the present survey was conducted using the questionnaire method, the depth of information able to be obtained is limited and may be more susceptible to social desirability bias. In future research, qualitative research methods would be valuable in offering greater depth of information to explore the issues which influence decision making and fear related to dementia. Further research targeted to a general community sample or longitudinal survey may also be of benefit to understanding attitudes and beliefs about dementia.

## Conclusions

The present study revealed that dementia is second only to cancer as the most feared disease. Findings suggest the need to provide education about the lesser-known impacts of dementia on physical functioning and life expectancy. Participants wanted to be informed about a diagnosis of dementia as soon as possible so they have more time to accept the diagnosis and to consider future plans. Our findings suggest that physicians should discuss the possibility of diagnosis and the implications of this with both patients and their support persons. Such discussions may include the support available following diagnosis and the opportunities to plan ahead, which may alleviate some fears around diagnosis.

## Data Availability

The datasets used and/or analyzed during the current study are available from the corresponding author on reasonable request.

## Abbreviation

CVD: Cerebrovascular disease

## References

[1] World Health Organization. Dementia [Internet]. 2019. Available from: https://www.who.int/news-room/fact-sheets/detail/dementia.

[2] Asada T. Epidemiology of Dementia in Japan. In: Neuroimaging Diagnosis for Alzheimer’s Disease and Other Dementias. 2017. p. 1–10.

[3] Statistical Bureau. Statistical Handbook of Japan 2019. [Internet]. 2019. Available from: https://www.stat.go.jp/english/data/handbook/c0117.html.

[4] United Nation. World Population Ageing 2019 Higlights [Internet]. 2019. Available from: https://www.un.org/en/development/desa/population/publications/pdf/ageing/WorldPopulationAgeing2019-Highlights.pdf.

[5] Dubois B, Padovani A, Scheltens P, Rossi A, Dell G (2016). Timely Diagnosis for Alzheimer’s Disease: A Literature Review on Benefits and Challenges. J Alzheimer’s Dis.

[6] Milne A (2010). Dementia screening and early diagnosis: The case for and against. Health Risk Soc.

[7] Koch T, Iliffe S (2010). Rapid appraisal of barriers to the diagnosis and management of patients with dementia in primary care: a systematic review. BMC Fam Pract.

[8] Bradford A, Kunik ME, Schulz P, Williams SP, Singh H, Mph J (2009). Missed and Delayed Diagnosis of Dementia in Primary Care Prevalence and Contributing Factors. Alzheimer’s Dis Assoc Disord.

[9] World Health Organization. Global Health Observatory (GHO) data. [Internet]. 2018. Available from: https://www.who.int/news-room/fact-sheets/detail/the-top-10-causes-of-death.

[10] Lang L, Clifford A, Wei L, Zhang D, Leung D, Augustine G (2017). Prevalence and determinants of undetected dementia in the community: a systematic literature review and a meta-analysis. BMJ Open.

[11] Low L, Swaffer K, Brodaty H (2019). Communicating a diagnosis of dementia: A systematic mixed studies review of attitudes and practices of health practitioners. Dementia.

[12] Hout HP, Van, Jansen DA, Stalman WA (2007). Do general practitioners disclose correct information to their patients suspected of dementia and their caregivers? A prospective observational study. Aging Ment Health.

[13] Abe M, Tsunawaki S, Matsuda M, Cigolle CT, Fetters MD, Inoue M (2019). Perspectives on disclosure of the dementia diagnosis among primary care physicians in Japan: A qualitatively driven mixed methods study. BMC Fam Pract.

[14] Carpenter BD, Xiong C, Porensky EK, Lee MM, Brown PJ, Coats M, Johnson DMJ (2008). Reaction to a Dementia Diagnosis in Individuals with Alzheimer’s Disease and Mild Cognitive Impairment. J Am Geriatr Soc.

[15] Umegaki H, Onishi J, Suzuki Y, Endo H, Iguchi A (2007). Attitudes toward disclosing the diagnosis of dementia in Japan. Int Psychogeriatrics.

[16] Lam TP, Sun KS, Chan HY, Lau CS, Lam KF, Robert S-F (2019). Perceptions of Chinese Towards Dementia in Hong Kong — Diagnosis, Symptoms and Impacts. Int J Environ Res Public Health.

[17] Watson R, Bryant J, Sanson-fisher R, Mansfield E, Evans T (2018). What is a ‘timely’ diagnosis ? Exploring the preferences of Australian health service consumers regarding when a diagnosis of dementia should be disclosed. BMC Health Serv Res.

[18] Jung JH, Kim MJ, Choi SH, Han NY, Park JE, Park HY (2017). Do patients want to listen to a diagnosis of dementia in Korea? Preferences on disclosing a diagnosis of dementia and discussing advance care planning in elderly patients with memory concerns and their families. Psychiatry Investig.

[19] Lin KN, Liao YC, Wang PN, Liu HC (2005). Family members favor disclosing the diagnosis of Alzheimer’s disease. Int Psychogeriatrics.

[20] Paul D, Wallis M, Henskens F, Nolan K. QuON A Generic Platform for the Collation and Sharing of Web Survey Data. 2013;111–6. Available from: https://ogma.newcastle.edu.au/vital/access/manager/Repository/uon:15474.

[21] R core team (2017). A language and environment for statistical computing.

[22] Alzheimer’s A. 2018 Alzheimer’s disease facts and figures. Alzheimer’s Dement. 2018;14:367–429.

[23] Yoshida D, Ohara T, Hata J, Shibata M, Hirakawa Y, Honda T (2020). Lifetime cumulative incidence of dementia in a community-dwelling elderly population in Japan. Neurology.

[24] Ebert A, Kulibert D, Mcfadden SH. Effects of dementia knowledge and dementia fear on comfort with people having dementia: Implications for dementia-friendly communities. Dementia. 2019;1–13. 10.1177/1471301219827708 30739490

[25] Kazui H, Harada K, Eguchi YS, Tokunaga H, Endo H, Takeda M (2008). Association between quality of life of demented patients and professional knowledge of care workers. J Geriatr Psychiatry Neurol.

[26] Alzheimer’s Disease International. World Alzheimer Report 2019: attitudes to dementia. Alzheimer’s Disease International: The International Federation of Alzheimer’s Disease and Related Disorders Societies, Inc. 2019.

[27] Lopez RP, Rose KM, Kenney L, Sanborn V, Davis JD (2020). Managing Shame: A Grounded Theory of How Stigma Manifests in Families Living With Dementia. J Am Psychiatr Nurses Assoc.

[28] Umegaki H, Suzuki Y, Ohnishi J, Iguchi A (2009). Changes in the perception of dementia in Japan. Int Psychogeriatrics.

